# Heart failure classification using deep learning to extract spatiotemporal features from ECG

**DOI:** 10.1186/s12911-024-02415-4

**Published:** 2024-01-15

**Authors:** Chang-Jiang Zhang, Yuan-Lu, Fu-Qin Tang, Hai-Peng Cai, Yin-Fen Qian, Chao-Wang

**Affiliations:** 1 Taizhou Central Hospital, Affiliated Hospital of Taizhou University, Taizhou, China; 2 School of Electronic and Information Engineering (School of Big Data Science), Taizhou University, Taizhou, China; 3 College of Physics and Electronic Information Engineering, Zhejiang Normal University, Jinhua, China

**Keywords:** Heart failure, MIMIC- III, Deep learning, CNN-LSTM-SE model

## Abstract

**Background:**

Heart failure is a syndrome with complex clinical manifestations. Due to increasing population aging, heart failure has become a major medical problem worldwide. In this study, we used the MIMIC-III public database to extract the temporal and spatial characteristics of electrocardiogram (ECG) signals from patients with heart failure.

**Methods:**

We developed a NYHA functional classification model for heart failure based on a deep learning method. We introduced an integrating attention mechanism based on the CNN-LSTM-SE model, segmenting the ECG signal into 2 to 20 s long segments. Ablation experiments showed that the 12 s ECG signal segments could be used with the proposed deep learning model for superior classification of heart failure.

**Results:**

The accuracy, positive predictive value, sensitivity, and specificity of the NYHA functional classification method were 99.09, 98.9855, 99.033, and 99.649%, respectively.

**Conclusions:**

The comprehensive performance of this model exceeds similar methods and can be used to assist in clinical medical diagnoses.

## Introduction

Heart failure is a syndrome with complex clinical manifestations. It can occur for a variety of reasons, including structural damage to the heart and changes in its function that prevent it from pumping blood to the body correctly, leaving the body without full circulation. As our population ages, the number of patients with heart failure increases yearly, with repeated hospitalization, reduced quality of life, and other problems. These problems highlight the need for timely diagnosis, treatment, and prognosis. Estimating the severity of patients with heart failure through its classification has important clinical significance in effective treatment.

Classifying heart failure is considered the most crucial step in treating it. The standard for classifying heart failure severity is the New York Heart Association (NYHA) functional classification [1], which pays attention to patients’ exercise habits and disease symptoms. NYHA Class I indicates that the patient with heart disease is physically active. NYHA Class II indicates the patient is somewhat limited in physical activity, engages in daily activities, but has begun to experience structural changes in the heart. NYHA Class III indicates the patient is significantly limited in physical activity, engages in little daily activity, and has significant structural changes in the heart. NYHA Class IV indicates that the patient cannot do any physical activity and has a considerable structural change in the heart.

The electrocardiogram (ECG) is used to monitor heart health by detecting the heart’s change, which can provide a clinical reference to physicians simply and intuitively [2]. There are many differences between the ECG signals (ECGs) from patients with heart failure and ordinarily healthy people. The grading of heart failure requires careful study of ECG recordings by experienced cardiologists, a process that is tedious and time-consuming. In addition, there may be small changes in the ECG that are ignored by the naked eye. Therefore, computer-aided diagnosis (CAD) algorithms [3] can be used to improve the accuracy of diagnosis. CAD uses machine learning [4] and deep learning methods to diagnose and analyze diseases from large-scale electronic medical data [5, 6]. For example, Balasubramanian et al. [7] used a method by combining convolutional neural network and support vector machine to segment retinal blood vessels. CAD can provide valuable reference results for medical personnel, reduce the workload of doctors, and help to reduce the occurrence of misdiagnosis to a certain extent.

Many researchers have used ECGs to study the classifications of heart failure. Tripoliti et al. [8] dealt with the severity of heart failure as a second-, third-, and fourth-level classification problem. Eleven classifiers were used on a heart failure dataset of 378 patients via 10-fold cross-validation and evaluated. The highest detection accuracy for the secondary, tertiary, and quaternary classification problems was 97, 87, and 67%, respectively. Zhang et al. [9] constructed datasets of patients with heart failure. Natural language processing (NLP) was used according to the relevant data on NYHA classification to classify patients with heart failure from clinical data (NYHA Classes I–IV). Qu et al. [10] extracted multiple features from the heart rate variability (HRV) of patients with heart failure. Support vector machine (SVM) and classification and regression tree (CART) were used to distinguish patients with heart failure with NYHA class I–III according to extracted features. The accuracy, sensitivity, and specificity of the SVM classifier reached 84.0, 71.2, and 83.4%, respectively, while the accuracy, sensitivity, and specificity of the CART classifier reached 81.4, 66.5, and 81.6%, respectively. Li et al. [11] proposed a deep convolutional neural network recursive neural network (CNN-RNN) model for real-time automatic classification of heart failure. Features of ECGs were extracted and combined with other clinical features. The combined features were provided to the RNN for classification, resulting in five classification results (typical and NYHA Classes I–IV). The proposed CNN-RNN model has a classification accuracy of 97.6%, sensitivity of 96.3%, and specificity of 97.4%. Li et al. [12] divided ECGs into 2 s segments and proposed a new multi-scale residual network (ResNet) to distinguish heart failure patients with different NYHA classes (NYHA Classes I–IV). The experimental results showed that the average positive predictive value, sensitivity, and accuracy of the proposed ResNet-34 were 93.49, 93.44, and 93.60%, respectively. D’Addio et al. [13] extracted features from Poincaré plot,which was generated from 24 h ECG recordings. They used machine learning algorithms to distinguish heart failure patients with different NYHA classes (NYHA Classes I–III). The machine learning algorithms used by the author included AdaBoost, k-Nearest neighbors (KNN), and naive Bayes (NB). The accuracy of the three algorithms was greater than 80%, and the area under the receiver operating curve was greater than 0.7. Sandhu et al. [14] analyzed 13 clinical medical data records on 299 patients with heart failure and classified these patients as NYHA Class III or IV. The SVM-GA model was proposed to classify the grade of patients with heart failure and calculate the importance of features. The accuracy, positive predictive value, and recall of the proposed SVM-GA model were 91.49, 94.25, and 93.6%, respectively. Tsai and Morshed [15] used BIDMC congestive heart failure (CHF) datasets, including the ECG of NYHA Class III and IV patients. Twenty-eight features were extracted from the ECG data. Machine learning models (including SVM, KNN, ensemble tree, decision tree, naive Bayes, and logistic regression) were used to realize automatic real-time, high-precision classification of patients. KNN was the most accurate, with 99.4% accuracy; the accuracy of SVM, ensemble tree, decision tree, naive Bayes, and logistic regression was 99.4, 98.2, 99.4, 98.7, and 99.2%, respectively.

**Fig. 1:**
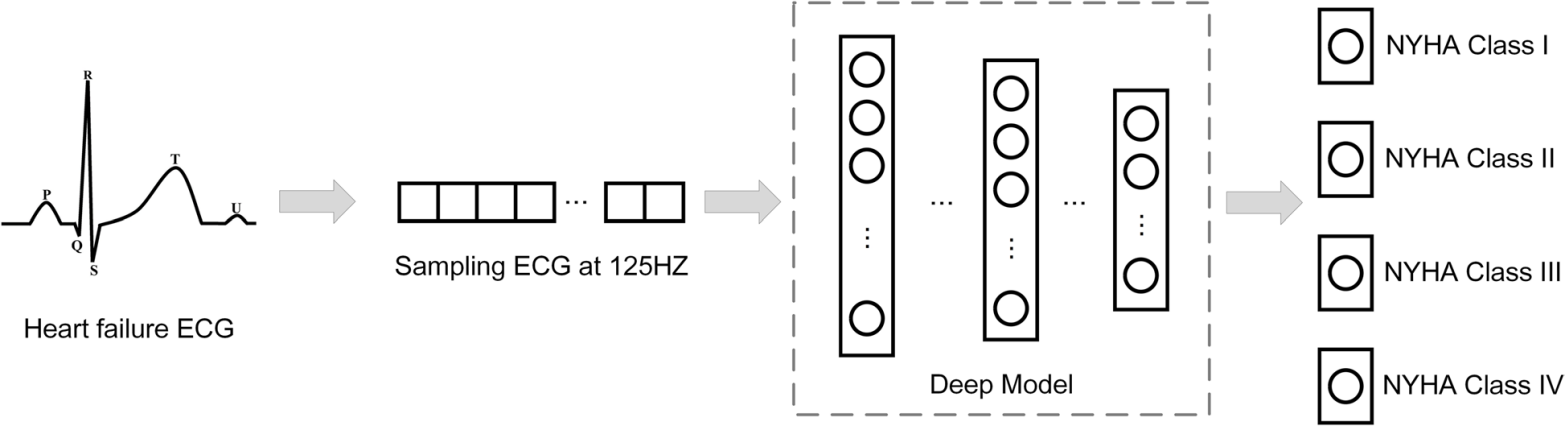
The framework of our method

The above studies showed that the severity of heart failure is primarily based on the NYHA classification standard. In comparison, few studies classified heart failure into four categories. Zhang et al. [9] and Sandhu et al. [14] used the patients’ medical data as the datasets, and D’Addio et al. [13] used the Poincaré chart as their experimental data. ECG or HRV [16] was used as experimental data in other literatures [8, 10–12, 15]. This demonstrates that many kinds of computer data are used in the research of heart failure grading and that there is no universal automatic assessment model of heart failure yet. Therefore,we studied an objective and convenient heart failure classification model, which only uses ECGs to evaluate the severity of heart failure. Our model is essentially a multi-classification task, and the framework of our model is shown in Fig. 1. The model can classify the severity of heart failure of patients, and the higher the NYHA grade represents the higher the severity of heart failure. The specific details about the proposed deep learning model of Fig. 1 are elaborated in Section III.

The main contributions in this paper are as follows:

1. Construct a deep learning model for heart failure classification using CNN and Long short-term memory (LSTM) to extract the spatial and temporal characteristics of the ECGs of patients with heart failure, and incorporate the attention mechanism to make the model focus on the key features of ECGs in patients with heart failure automatically.
2. The CNN-LSTM-SE model proposed in this paper has the characteristics of simple structure and lightweight. Noise filtering, feature extraction and selection techniques are not required.
3. Discuss the effect of different length ECGs of patients with heart failure on heart failure classification, and find out the best partition. Train and verify the performance of the proposed CNN-LSTM-SE deep learning model that automatically divides cases of heart failure into four categories according to the NYHA classification standard based on the best ECG segment signals of patients with heart failure.
4. Conduct an interpretability analysis of the proposed deep learning model, overlaying the ECG with the heat maps generated using Gradient-weighted Class Activation Mapping (Grad-CAM) for visualization. By comparing ECGs of 4 different severity grades of heart failure, it was observed that for NYHA Class I ECG, the proposed model mainly focus on the QRS segment. For NYHA Class II-IV heart failure, the proposed model’s attention is mostly concentrated on the ST-T segment. This has some indicative effect on the decision of the assistant clinician.
5. The proposed model in this paper has been tested on different datasets of heart failure and achieved good results, indicating that the proposed model has good robustness.

## Data

### Database

The Medical Information Mart for Intensive Care III (MIMIC - III) is an extensive, freely available database of health-related data associated with over 40,000 patients who stayed in critical care units of the Beth Israel Deaconess Medical Center between 2001 and 2012 [17]. MIMIC-III includes mainly clinical and waveform datasets. The clinical datasets contain 26 data tables, which record and store patient demographic information, vital signs, laboratory results, surgical information, medication, nursing records, in-hospital mortality, electronic medical records, and other information. The waveform data centrally record the patient’s ECG signal data, respiratory data, heart rate variability data, blood pressure data, and blood oxygen saturation data.

### Data-set establishment

**Fig. 2:**
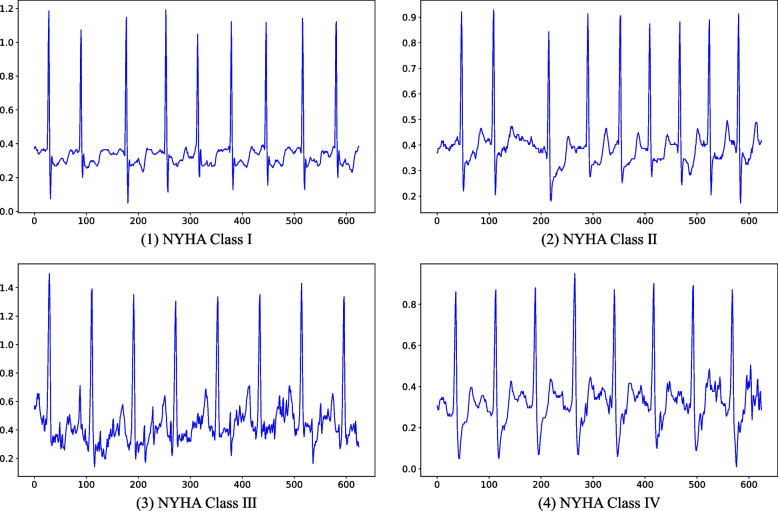
Example ECGs for different classes

Based on the MIMIC-III v1.4 database, heart failure classification is studied by combining deep learning with ECG signal. First, all ICD-9 codes relevant to heart failure was identified from the DIAGNOSES_ICD table within the data set. A total of 25 codes for heart failure conditions were found in the table, including: congestive heart failure, systolic heart failure, diastolic heart failure and so on. Patients’ diagnosis results were recorded in DRGCODES.csv file of the MIMIC-III data set. A total of 10,436 patients with heart failure were screened from DRGCODES.csv file according to ICD-9 coding, among which 644 patients with heart failure were labeled with NYHA grading results. Finally, by cross-referencing patient IDs, multi-lead ECG data was collected from the waveform data set for 268 heart failure patients. Not every one of these 268 patients had a complete multi-lead ECG. For data consistency, we used the lead II ECG as the data set for this article. The resulting severity grading distribution of heart failure is presented in Table 1, while examples of the ECGs of the four NYHA grades are shown in Fig. 2**,** the abscissa represents the sampling point and the ordinate represents the amplitude of the ECG.

**Table 1:** Data used in this study

Type	No. of Patients	Proportion
NYHA Class I	8	2.99%
NYHA Class II	47	17.54%
NYHA Class III	115	42.91%
NYHA Class IV	98	36.57%
Total	268	100%

Not every patient in the waveform datasets had ECG recordings, so there was an imbalance in the distribution of the datasets. To solve the problem of unbalanced data distribution, we adopted the method of setting initial weights, dividing the training set, and test set according to the data distribution proportions, and employing cross-validation [18].

### Pre-processing

**Fig. 3:**
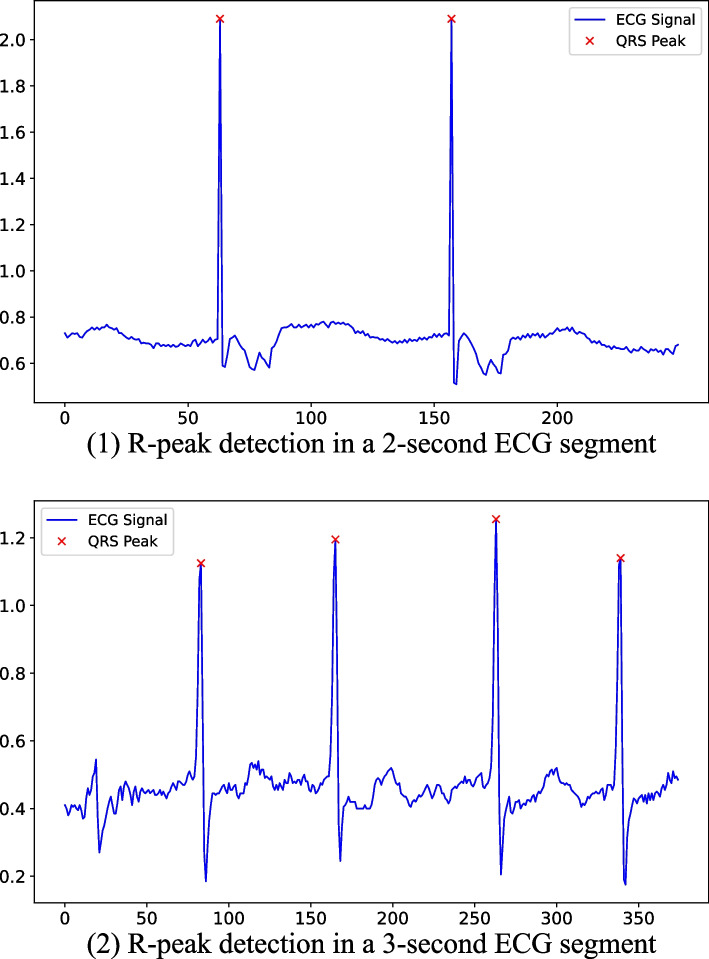
The results of R-peak detection

The data used in this study included 30 min lead II ECGs of patients with different heart failure grades, which needed to be segmented before they were entered into a deep learning network. The sampling frequency of the original ECG signal was 125 Hz. We used the original sampling frequency and recorded the whole ECG signal in segments of 2–20 s. Some studies indicate that irregular R-R intervals may indicate cardiac functional abnormalities [19]. To ensure that the proposed deep learning model captures information from continuous wave peaks, we performed R-peak detection on ECG segments of different durations for data preprocessing [19]. Segments without at least 2 R-peaks were excluded, ensuring that each segment contained at least two complete QRS waves. The algorithm involves dynamic threshold computation, peak detection, sliding window, and QRS wave validation. Figure 3 illustrates the R-peak detection results for 2-second and 3-second ECG segments, showing that Fig. 3(1) contains two complete QRS waves, while Fig. 3(2) contains four complete QRS waves. Similar results can be obtained for other durations in Table 2. Results for other durations are not presented here for brevity.

**Table 2:** Summary of the amounts of data segmented by different durations

Split seconds	Number
2 s	225,619
3 s	149,714
4 s	111,863
5 s	89,210
6 s	74,119
8 s	55,316
9 s	49,049
10s	44,055
12 s	36,563
15 s	29,099
18 s	24,122
20s	21,654

The amounts of data after performing R-peak detection for data cleaning on ECG segments of different durations are presented in Table 2.

Thirty minutes of ECGs could not be evenly segmented by 7, 11, 13, 14, 16, 17, and 19 s intervals, so they were excluded. We modeled and tested the datasets of the remaining ECG recordings to find the partitioning with the best effect.

Finally, to speed up the optimal gradient descent solution [20], we conducted Z-score standardization processing on the datasets. The formula is as follows:

1 $${x}^{\prime }=\frac{x_i-\mu }{\sigma },$$

where *x*^′^ represents the normalized ECG segments, *x*_*i*_ is the sampled ECG signal, *μ* is the mean, and *σ* is the variance of the population data.

## Deep learning model

### One-dimensional convolutional neural networks

Convolutional neural network (CNN) is feedforward neural network with deep structure, convolution calculation, and a representative deep learning algorithm [21]. The study of CNN began in the 1980s, LeNet-5 being one of the earliest [22]. After improved deep learning theory and computing equipment were introduced in the 2000s, CNN developed rapidly and were applied to computer vision, natural language processing, and other fields. Since the ECG datasets in this study are one-dimensional, unlike the two-dimensional image input to a standard CNN, we used a one-dimensional CNN for better results [11].

A one-dimensional CNN includes a one-dimensional convolution layer, a pooling layer, and a fully connected layer [21]. A one-dimensional CNN learns the spatial features of data automatically without artificial feature selection. Therefore, we used the CNN as a feature extractor. An ECG signal contains strong temporal characteristics, and a simple CNN cannot extract the features of temporal signals well. It must be combined with other deep learning networks that are good at processing temporal signals.

This study used a nine-layer deep CNN, including three one-dimensional convolution layers, three pooling layers, and three full connection layers. Adding a pooling layer behind the convolution layer reduces the feature map’s size, and the full connection layer outputs features for the final classification task.

### Long short-term memory

Long short-term memory (LSTM) is a type of recurrent neural network (RNN) that is often used to predict information containing time sequences [23]. RNN is connected to evaluating the current information based on the previous period’s data, so it performs well in predicting timing problems. However, an RNN is prone to gradient disappearance with increased network layers. Based on RNN, LSTM increased the screening of memory information, retained useful information for the model, and solved the RNN problem of gradient disappearance and explosion [24].

**Fig. 4:**
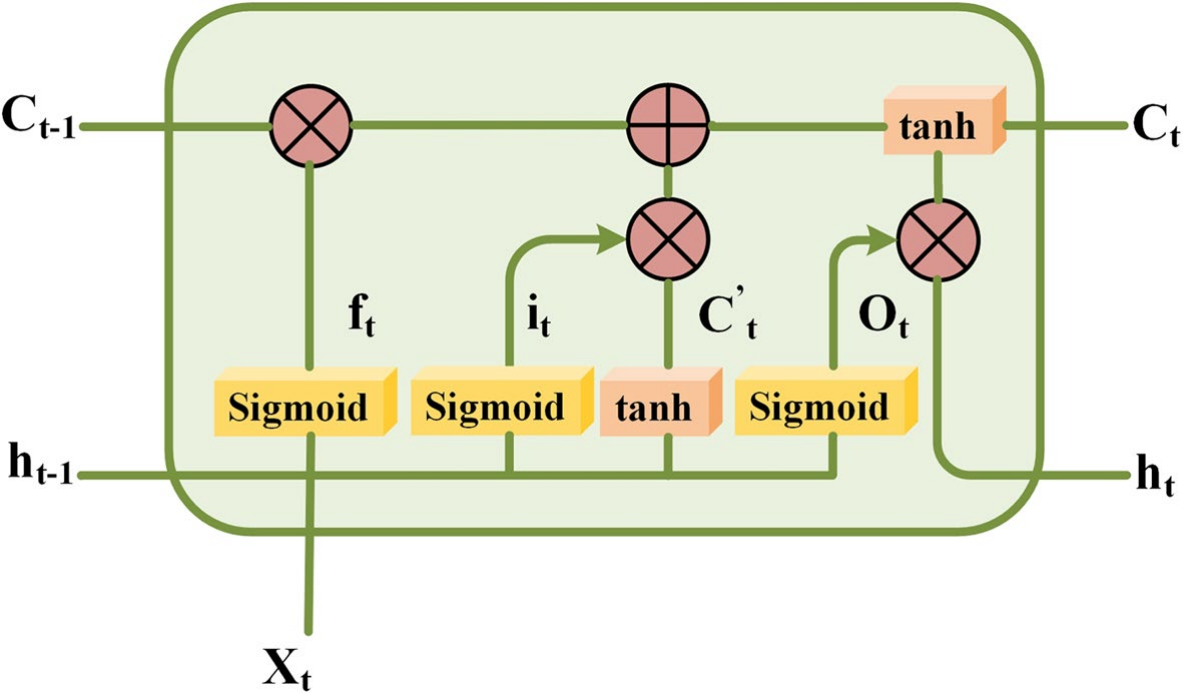
Internal structure of LSTM block

Figure 4 shows the internal structure of an LSTM memory block. *C*_*t*_ and *C*_*t* − 1_ are the neuronal states of the current moment and the previous moment, respectively. *h*_*t*_ and *h*_*t* − 1_ are respectively the output of the unit at the current time and the unit at the previous time, and *X*_*t*_ is the input to the network. The LSTM forget gate is *f*_*t*_, which controls forgotten information through the sigmoid function. *i*_*t*_ is the input gate, which sets the threshold value and implements the tanh function to determine the state of the neuron. *O*_*t*_ is the output gate, which controls the output information through the sigmoid function. The formulas are as follows:

2 $${f}_t= Sigmoid\ \left({K}_f\cdot \left[{h}_{t-1},{X}_t\right]+{Z}_f\right),$$

3 $${i}_t= Sigmoid\ \left({K}_i\cdot \left[{h}_{t-1},{X}_t\right]+{Z}_i\right),$$

4 $${O}_t= Sigmoid\ \left({K}_O\cdot \left[{h}_{t-1},{X}_t\right]+{Z}_O\right)$$

5 $${C}_t^{\prime }=\tanh \left({K}_c\cdot \left[{h}_{t-1},{X}_t\right]+{Z}_c\right)$$

where *K*_*f*_, *K*_*i*_, *K*_*o*_, and *K*_*c*_ represent the weight matrix corresponding to the amnesia gate, input gate, output gate, and neuron state matrix, respectively, and *Z*_*f*_, *Z*_*i*_, *Z*_*o*_, and *Z*_*c*_ represent the offset for each door.

The neuron’s current state and the cell’s output are expressed as follows:

6 $${C}_t={f}_t\cdot {C}_{t-1}+{i}_t\cdot {C}_t^{\prime }$$

and

7 $${h}_t={O}_t\cdot \tanh \left({C}_t\right).$$

### Channel attention module

A problem arises when training a neural network. With the deepening of network layers, the final classification effect decreases instead of increasing, and even the accuracy of the training set stagnates. This happens because although increasing the network layers may obtain deeper features, the network cannot select these features well. We integrate a channel attention mechanism into a CNN to amplify the features of a particular part while ignoring irrelevant features and fully using the existing convolutional layer without increasing the depth of the network.

**Fig. 5:**
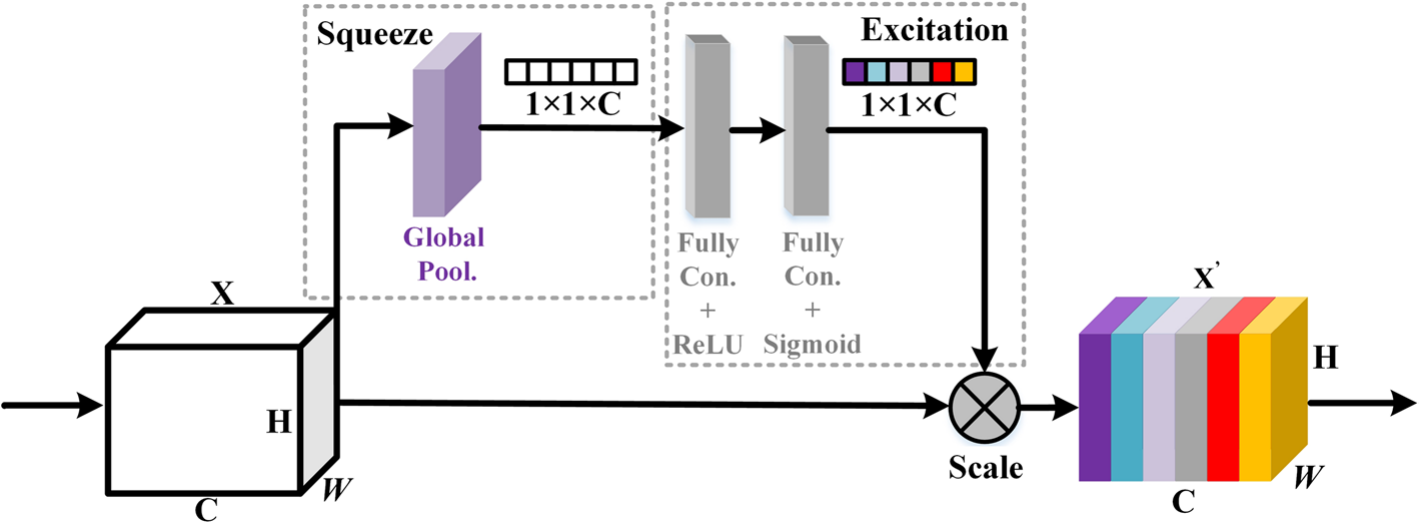
Internal structure of SE-Net

The squeeze-and-excitation network (SE-Net) [25] is a channel attention mechanism. It is a new image recognition structure unveiled by autonomous driving company Momenta in 2017. The modeling of the correlation between feature channels is the excitation network. The central ideas of SE-Net are to learn feature weights through the network according to a loss function, to enlarge the effective feature map weight, and to reduce invalid or small-effect feature map weights for better results. The internal structure of SE-Net is shown in Fig. 5**.** The first step of SE-Net is to change the elements in each channel into scalars through global average pooling, called Squeeze operation. The second step is to pass the scalar value through the two fully connected (FC) layers to obtain a weight between 0 and 1. The process obtains the new feature map by multiplying each element of the original H × W by the weight of the corresponding channel. This step is called excitation. Finally, channel-by-channel weighting recalibrates the original features in the channel dimension.

We added the SE-block after the second and third convolution layers of the CNN to automatically select related features and ignore irrelevant ones, resulting in a better classification of heart failure.

### CNN-LSTM-SE model integrating attention mechanism

**Fig. 6:**
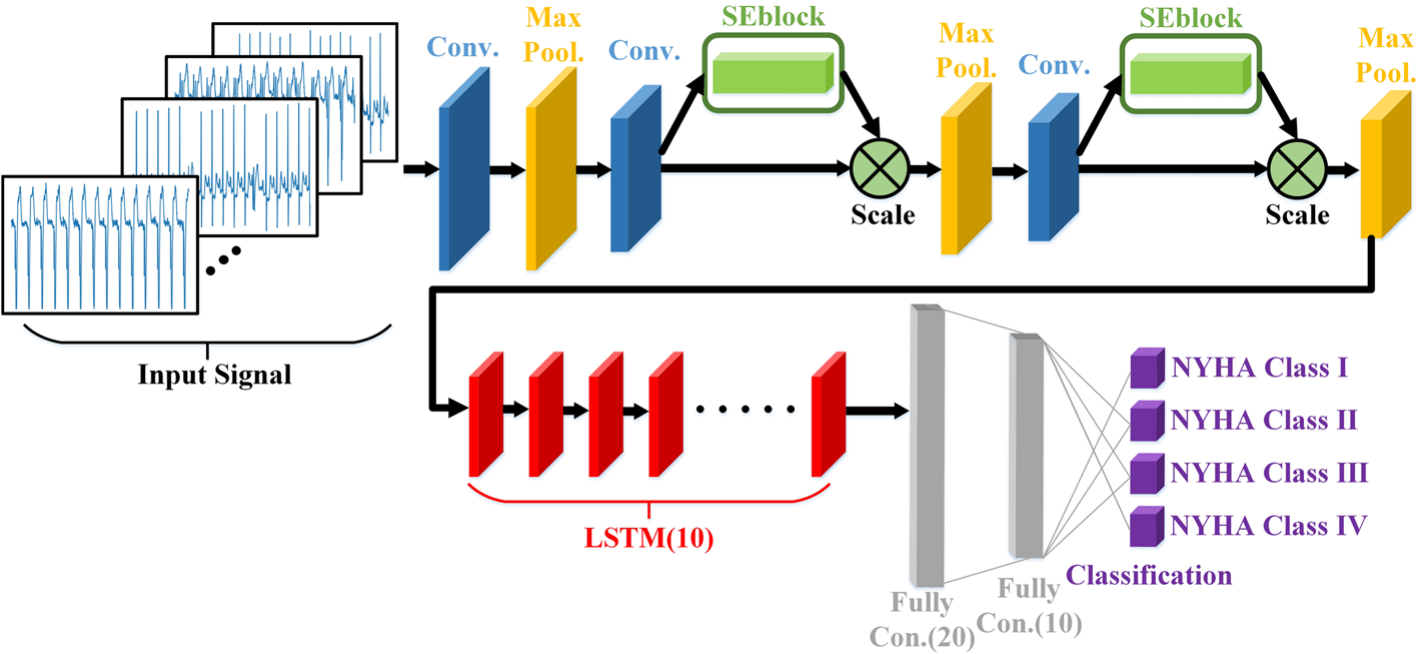
Architecture diagram of the proposed CNN-LSTM-SE model

The structure of our proposed CNN-LSTM-SE model with an integrated attention mechanism is shown in Fig. 6. We performed an ablation experiment [26] to determine the optimal network structure proposed in this paper. The proposed network contains 20 layers which includes 3 convolutional layers, 2 SE-Blocks, 10 LSTM layers, 3 global average pooling layers, and 2 fully connected (FC) dense layers. First, one-dimensional CNN was used to extract the spatial features of ECGs. Second, the LSTM layer was added before the FC layer of the CNN to make the model learn the sequential characteristics of the ECGs. Finally, the attention mechanism SE-block was added behind the second and third convolution layers of the CNN-LSTM model to realize automatic focusing of the relevant features and to ignore irrelevant features.

From one-dimensional CNN model to the CNN-LSTM model and finally to the CNN-LSTM-SE model, the accuracy, specificity, sensitivity, and positive predictive value were successively improved. The CNN-LSTM-SE model provided the best results, which shows that the integration of LSTM and attention mechanism in one-dimensional CNN model can improve the effect of heart failure classification. The test results of three models are described in Section V.

## Implementation details

The software environment for this experiment was Tensorflow2.3.0 and Python 3.8, and the hardware environment was an NVIDIA GeForce GTX 1060.

A five-fold cross-validation method was adopted to evaluate the robustness of the proposed model [27]. This method divided the datasets randomly into five parts, four of which were trained and one tested. The cycle was repeated five times to build five models. Datasets divided into 2–20 s segments were modeled separately. Twelve modeling test results are described in Section V. The evaluation indexes of each fold were accuracy, sensitivity, and specificity. Finally, the accuracy, sensitivity, specificity, and positive predictive value of the five models were averaged to get the final evaluation index results. The average training time for each model is 226 seconds, and the total training time for five-fold cross-validation is 18 minutes. The average time taken for model testing is 0.65 seconds.

We chose the Adam optimizer with backpropagation, set the learning rate of 0.001 for each round of training fold, trained for 60 epochs, and set the maximum mass size to 32.

## Results and discussion

For unbalanced samples, using only accuracy did not help to comprehensively evaluate the model’s performance. Therefore, four objective standard indexes were used to evaluate the classification performance of the proposed mode: accuracy (Acc), positive predictive value (PPV), specificity (Spe), and sensitivity (Sen). Acc, PPV, Spe, and Sen are defined as follows (true positive [TP], false positive [FP], true negative [TN], and false negative [FN] are used in the formula):

Acc refers to the percentage of predicted correct results of the total samples:

8 $$\textrm{Acc}=\frac{TP+ TN}{TP+ TN+ FP+ FN}.$$

PPV refers to the probability of actual positive samples among all predicted positive samples:

9 $$\textrm{PPV}=\frac{TP}{TP+ FP}.$$

Spe refers to the probability of being predicted as a negative sample in the actual negative samples:

10 $$\textrm{Spe}=\frac{TN}{TN+ FP}.$$

Sen refers to the probability of being predicted as a positive sample in the actual positive sample:

11 $$\textrm{Sen}=\frac{TP}{TP+ FN}.$$

**Fig. 7:**
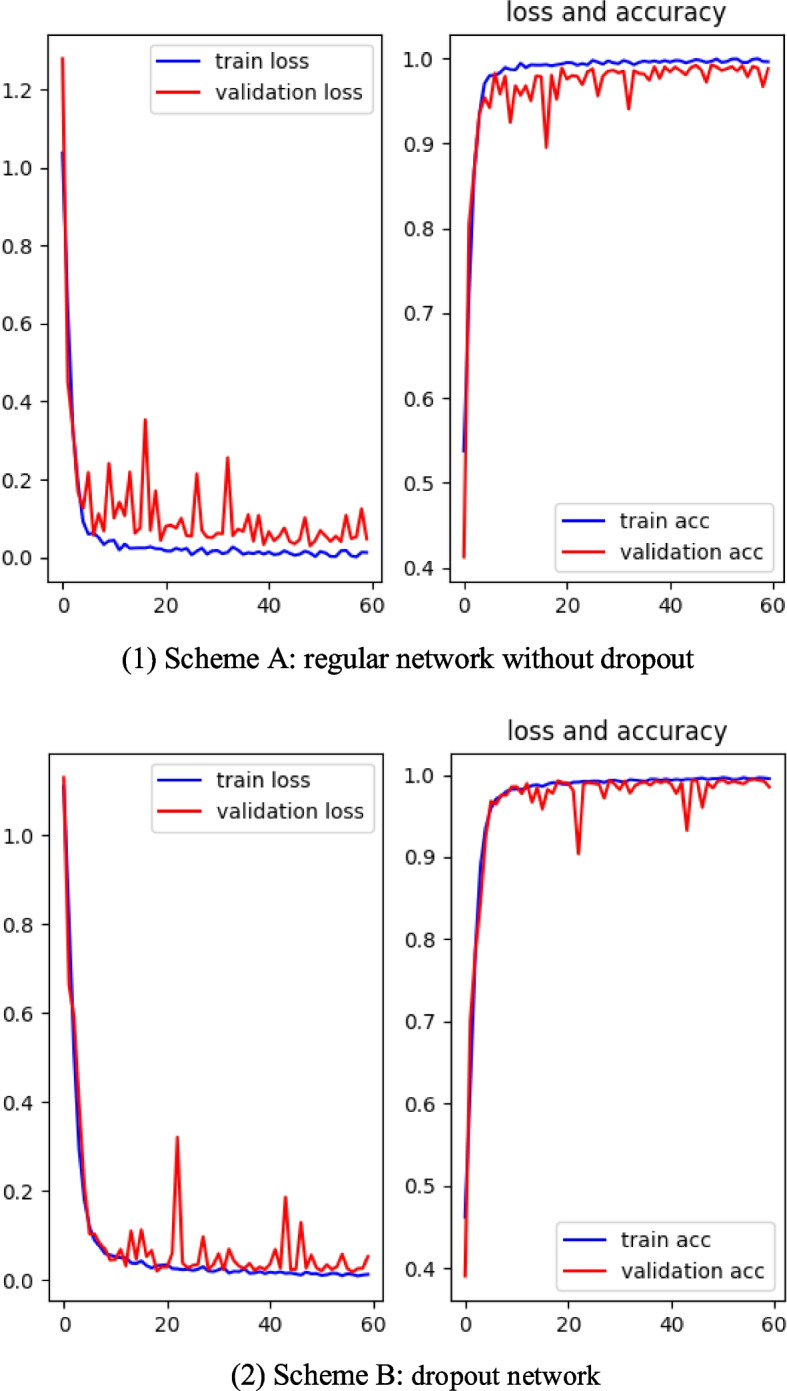
Accuracy and loss plots for the various schemes during training

We adopted two kinds of schemes in the training. Scheme A is a trained network without any dropout and is introduced as reference to examine the effect between a regular network and dropout network. The other is dropout scheme. In Scheme B, 20% of the recurrent and input connections of the LSTM layer are dropped out. The accuracy and loss curves for each of these schemes are presented in Fig. 7**.** It can be observed from Fig. 7 that the dropout network has little fluctuation in the accuracy curve compared to the regular network. Both the validation curve and the training curve steadily increase and eventually stabilize at around 99% at 60 epochs. The validation set loss curve of the conventional network oscillates significantly. At 60 epochs, the accuracy of the training set stabilizes at 99%, while the accuracy of the validation set is 98%. The accuracy of the validation set of the Scheme A is 1% lower than that of the Scheme B.

The test results of three models (CNN, CNN-LSTM, CNN-LSTM-SE) generated by the ablation experiment are shown in Table 3. The datasets used were patients’ ECGs divided into 12 s segments. Table 3 shows that by adding the LSTM layer to the CNN (CNN-LSTM model), the Acc, PPV, Sen, and Spe of the model increase by 0.69, 1.441, 0.4165, and 0.2155%, respectively. By incorporating the attention mechanism into the CNN-LSTM model (CNN-LSTM-SE model), the Acc, PPV, Sen, and Spe of the model increase by 0.452, 0.3845, 0.7795, and 0.1835%, respectively.

**Table 3:** Comparison of different model performance on ECG datasets divided by 12 s

Model	Acc(%)	PPV(%)	Sen(%)	Spe(%)
CNN	97.948	97.16	97.837	99.25
CNN-LSTM	98.638	98.601	98.2535	99.4655
CNN-LSTM-SE	99.09	98.9855	99.033	99.649

Twelve datasets, divided into 2–20 s intervals, were modeled separately. The results of 12 CNN-LSTM-SE network modeling tests incorporating an attention mechanism are shown in Table 4. The accuracy, positive predictive value, sensitivity, and specificity of the model divided into 12 s segments are 99.09, 98.9855, 99.033, and 99.649%, respectively. Compared with other segmentation methods, this model (12 s segments) has the highest accuracy, positive predictive value, specificity, and third-highest sensitivity. The sensitivity of the model divided by 12 s sementation is 0.001% lower than that divided by 9 s segmentation (ranking second), and 0.077% lower than that divided by 15 s segmentation (ranking first). The sensitivity of the model divided by 12 s segmentation is almost equal to that of the second best. Therefore, the proposed CNN-LSTM-SE model has the best comprehensive performance when the datasets are divided into one segment every 12 s.

**Table 4:** Performance comparison of CNN-LSTM-SE model on ECG datasets divided by different durations

Split seconds	Acc(%)	PPV(%)	Sen(%)	Spe(%)
2 s	97.472	97.471	97.426	99.043
3 s	97.748	97.4675	97.936	99.1595
4 s	98.114	97.891	98.254	99.2805
5 s	98.332	98.356	98.4665	99.3655
6 s	98.65	98.491	98.7575	99.4845
8 s	**98.942**	**98.967**	98.9565	**99.5945**
9 s	98.812	98.7465	**99.034**	99.5525
10s	98.706	98.917	98.704	99.4965
12 s	**99.09**	**98.9855**	**99.033**	**99.649**
15 s	**98.938**	**98.937**	**99.11**	**99.597**
18 s	98.778	98.433	98.682	99.5405
20s	98.37	97.969	97.7495	99.4055

Bold font indicates the top three best evaluation parameters

**Fig. 8:**
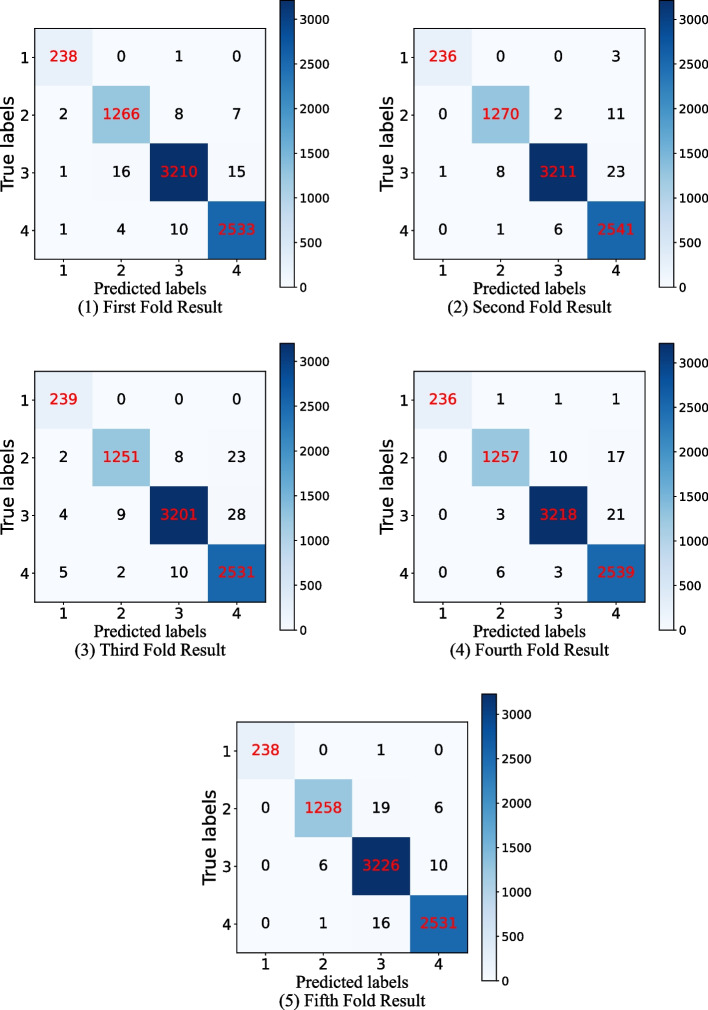
The confusion matrixes of the CNN-LSTM-SE model divided into one segment by every 12 s

The confusion matrixes of the CNN-LSTM-SE model divided into 12 s segments are shown in Fig. 8. As shown in Fig. 8, the model is more likely to confuse all grades of heart failure with those of neighboring grades, and less likely to confuse those of different grades. For example, in Fig. 8(1), 16 patients with NYHA Class III heart failure were misclassified as NYHA Class II, 15 cases were misclassified as NYHA Class IV, and only 1 case was misclassified as NYHA Class I. In Fig. 8(5), 16 patients with NYHA Class IV heart failure were misclassified as NYHA Class III and only 1 was misclassified as NYHA Class II. This suggests that there is greater similarity between adjacent grades of heart failure ECGs than that of different grades, making the models difficult to distinguish.

The model test results for the five-fold cross-validation are shown in Table 5. Table 5 shows that, except for the third fold model, the Acc is 98.76%, and the classification effect is slightly poor. The Acc of the other-fold heart failure grade classification models is above 99%. The average PPV was 98.9855%, close to 99%, the average Sen was 99.033%, and the average Spe was 99.649%, close to 100%. It indicates that the model divided by 12 s segmentation is relatively excellent in all indicators.

**Table 5:** Five-fold cross-validation of CNN-LSTM-SE model

Fold	Acc(%)	PPV(%)	Sen(%)	Spe(%)
First Fold	99.11	98.835	99.17	99.67
Second Fold	99.25	99.2975	99.1175	99.715
Third Fold	98.76	98.0475	98.875	99.5325
Fourth Fold	99.14	99.3175	98.8875	99.665
Fifth Fold	99.19	99.43	99.1175	99.6625
Average	99.09	98.9855	99.033	99.649

To further verify the performance of the proposed CNN-LSTM-SE model, we tested the performance of our model on two other datasets (Data-sets A and B). The Data-set A were obtained from public datasets (PhysioBank) namely the Beth Israel Deaconess Medical Centre (BIDMC) Congestive Heart Failure Database [28] and Fantasia Database [29]. The Data-set B was obtained from the Intercity Digital ECG Alliance (IDEAL) study of the University of Rochester Medical Center Telemetric and Holter ECG Warehouse (THEW) archives [30]. The details of ECG signals obtained from various databases is presented in Table 6. The BIDMC database contains ECGs from 15 patients with CHF, classified according to the NYHA classification standard, without distinguishing between NYHA classes III and IV. The Fantasia database includes ECGs from 18 healthy individuals. The THEW database contains ECGs from 50 patients with CHF, categorized into 1–4 severity grades, although the classification standard used for this categorization are not explicitly stated.

**Table 6:** The details of ECG signals obtained from various databases

Database	Diagnosis	Number of ECG records	Number of 12 seconds ECG segments
BIDMC	CHF	15 (NYHA III-IV)	22,500
Fantasia	Normal	18	46,380
THEW	CHF	50 (Severity of CHF Treatment 1–4)	7500

We used Data-set A (BIDMC + Fantasia) to perform a binary test for diagnosis of heart failure in patients with our model, and Data-set B (THEW) to perform a separate four-class classification test for assessment of heart failure severity in patients with our CNN-LSTM-SE model alone. The results are shown in Table 7. From Table 7, it can be seen that the binary classification model using Data-set A achieved an accuracy of 99.35%, precision of 99.35%, sensitivity of 99.37%, and specificity of 99.37%. The four-class classification model using Dataset B achieved the Acc of 98.91%, PPV of 98.39%, Sen of 99.06%, and Spe of 99.57%. Except for the Acc (98.91%) and PPV (98.39%) of the model using Data-set B, all other metrics of the proposed models constructed using Data-sets A and B are above 99%. The CNN-LSTM-SE model proposed in this paper also performs well on above two datasets, indicating that our model has strong robustness.

**Table 7:** Results on the data-sets A and B

Data-set	Acc(%)	PPV(%)	Sen(%)	Spe(%)
Data-set A (BIDMC+Fantasia)	99.35	99.35	99.37	99.37
Data-set B (THEW)	98.91	98.39	99.06	99.57

To further verify the performance of the proposed CNN-LSTM-SE model, the proposed model is compared with other existing heart failure classification methods (e.g. SVM, CNN, Natural Language Processing(NLP), Resnet, etc.). The performance indicators of each model are shown in Table 8. The current research on the classification of heart failure mainly includes two-, three-, four-and five-grades classification. Traditional shallow machine learning methods (e.g. SVM, CART, Adaboost, etc.) are mostly used to model the two-grades and three-grades studies of heart failure classification. However, the limitations inherent in shallow machine learning, such as manual feature extraction and inherent model characteristics, make it difficult to achieve high accuracy rates in heart failure classification. The Acc of the heart failure classification of the machine learning model is around 80–90%, which is generally about 10% lower than that of our CNN-LSTM-SE model. For the fourth-grades and five-grades heart failure classification problems, almost all the models are constructed by deep learning methods. For the four-grades heart failure classification problem, Zhang et al. [9] adopted the NLP method, and the patient’s clinical data was used as the input of the model. The Ppv of the model was 94.99%. Li et al. [12] improved ResNet-34 by adding multi-scale residual block to the Resnet-34. The Acc of heart failure classification obtained by the above model reached 94.29%, and the Ppv was 94.16%. Most heart failure classification techniques using deep learning largely rely on CNN for extracting the spatial features of ECG, neglecting the temporal characteristics. This paper presents an alternative method that incorporates LSTM to capture sequential features of ECG signal and the attention mechanism to focus important features associated with heart failure. Therefore, the effect of our CNN-LSTM-SE model is better than that of literature [9] and literature [12]. For the five-grades heart failure classification problem, the Acc of heart failure classification obtained by the CNN-RNN [11] model was 97.6%. The model focuses on both temporal and spatial features of the ECG, but the method proposed in this paper incorporates attention mechanisms to make the model more focused on key features related to heart failure, so the performance of our CNN-LSTM-SE model is better than the CNN-RNN model. The literature [11] only discussed the effect of dividing ECG according to 2 s and 5 s, while we discusses the impact of varying ECG segment lengths on heart failure classification and reveals that the 12 s ECG segment results in optimal accuracy. Our model is designed to tackle the four-grades heart failure classification problem, has yielded noteworthy results.

**Table 8:** Summary of performance comparison for different methods

Classification problem	Model	Number of data	Performance
Two classes	SVM-GA [14]	Clinical Data NYHA class III: 1365 NYHA class IV: 2522	Acc – 91.49% Ppv – 94.25% Recall–93.60%
	11-layer CNN [31]	5-seconds ECG segment CHF: 30000 Normal: 70308	Acc – 98.97% Sen – 98.87% Spe – 99.01%
Three classes	CART [10]	RR interval segment (*N* = 300) NYHA class I: 1416 NYHA class II: 3088 NYHA class III: 6181	Acc – 81.40% Sen – 66.50% Spe – 81.60%
	AdaBoost [13]	Poincaré plot NYHA class I: 22 NYHA class II: 116 NYHA class III: 61	Acc – 82.5% Ppv – 77.8% Sen – 58.3% Spe – 92.9%
Four classes	NLP [9]	Clinical note NYHA class I: 1367 NYHA class II: 2502 NYHA class III: 1790 NYHA class IV: 515	Ppv – 94.99% Recall–92.10%
	Multi-scale ResNet-34[12]	5-seconds ECG segment NYHA class I: 3720 NYHA class II: 7440 NYHA class III: 11940 NYHA class IV: 6240	Acc – 94.29% Ppv – 94.16% Sen – 93.79% Spe – 97.89%
	Our work	12-seconds ECG segment NYHA class I: 1200 NYHA class II: 7050 NYHA class III: 17250 NYHA class IV: 14700	Acc – 99.09% Ppv – 98.98% Sen – 99.03% Spe – 99.64%
Five classes	CNN-RNN [11]	2-seconds ECG segment Normal: 5160 NYHA class I: 2520 NYHA class II: 4680 NYHA class III: 3150 NYHA class IV: 6240	Acc – 97.60% Ppv – 97.10% Sen – 96.30% Spe – 97.40%

**Fig. 9:**
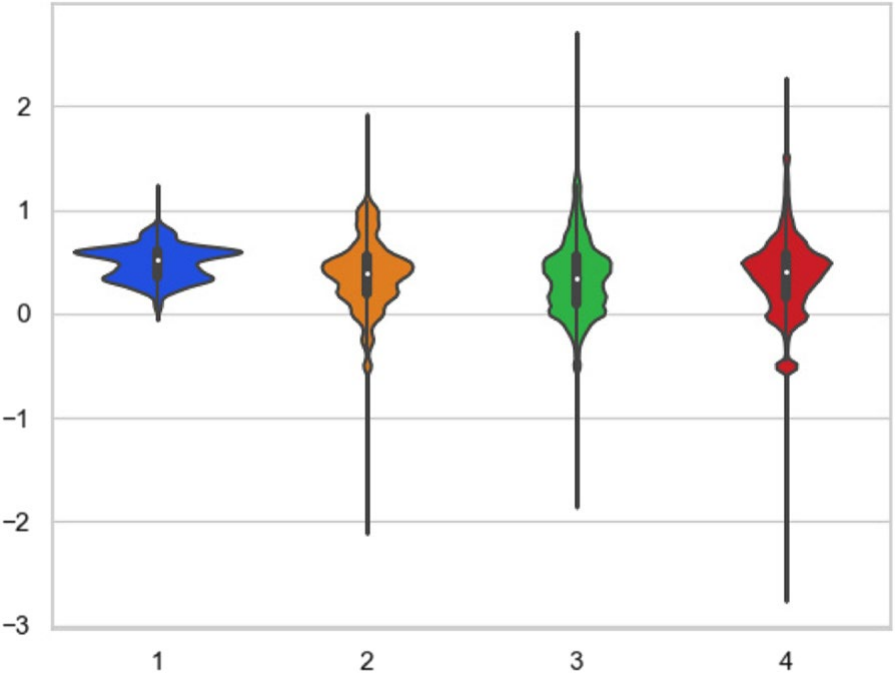
Violin diagram of ECG amplitudes for four severe levels of heart failure

We analyzed the data used in this experiment and visualized the results of ECG signal analysis. The violin diagram [32] of the ECG amplitude for each severity level of heart failure is shown in Fig. 9. The amplitude distribution of ECGs according to the severity of heart failure is more intuitively understood by observing the violin diagram. As shown in Fig. 9, the ECG signal amplitudes of NYHA Class I are all concentrated between 0 and 1. The amplitudes of the ECGs of NYHA Classes II, III, and IV are relatively dispersed, with the amplitudes of the ECGs of NYHA Class II being between − 2 and 2, of NYHA Class III being between − 2 and 2.8, and of NYHA Class IV being between − 2.8 and 2.2. However, the amplitudes of ECGs of NYHA Classes II, III, and IV are mainly concentrated between 0 and 1, except for a few distributed outliers. The distribution of the four categories is similar, with the maximum distribution around 0.5 and the number of distributions gradually decreasing to 0 and 1. In this case, some simple characteristics, such as amplitude, cannot be relied on to distinguish the type of heart failure. Therefore, building a deep learning model to distinguish between the four levels is necessary.

**Fig. 10:**
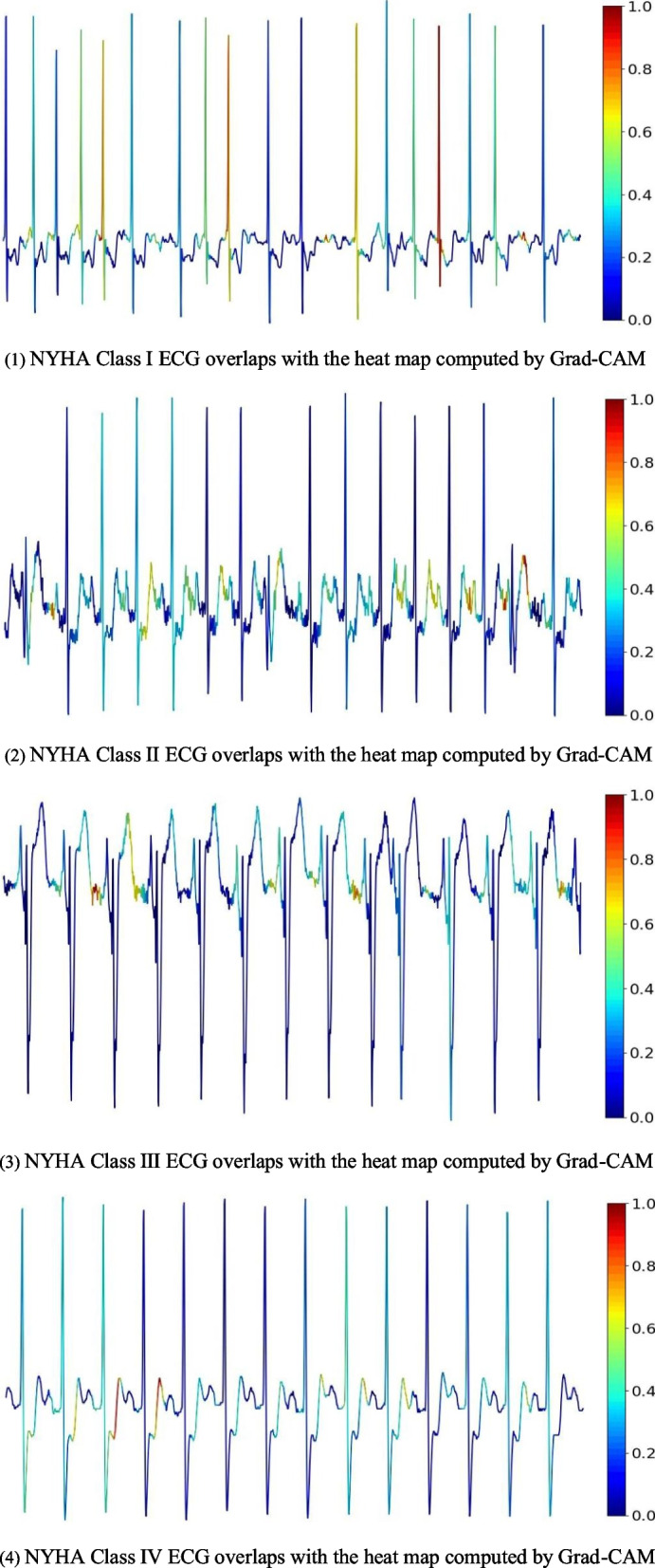
Visual interpretation of the CNN-LSTM-SE model

In addition, to enhance the interpretability of our model, we applied gradient-weighted class activation mapping (Grad-CAM) to obtain the heat maps of the last convolutional layers to highlight the area of the model’s focus. To visualize them, we displayed the heat maps for all four grades of heart failure. Figure 10 shows the heat maps of ECGs in heart failure NYHA Class I-IV, which are overlaid with heat maps of the last convolution layer calculated by the Grad-CAM method. The color bar ranging from blue to red indicating the degree of model attention, from low to high. From Fig. 10(1), it can be observed that the model focuses on the QRS of the ECG. Moreover, in Fig. 10(2)–(4), it is evident that the model predominantly concentrates on the ST segment of the ECG, which is known to exhibit abnormal changes in the ECG of heart failure patients [33]. As the disease progresses, the changes in the ST-T segment (the region of the ST and T waves) become more pronounced, which has a strong correlation with the severity of heart failure and serves as a reliable indicator. We can see that the ST-T segment of most ECGs is more red than other segments, and the results show that the model pays more attention to the ST-T segment location of the characteristic ECGs, which has some indicative effect on the decision of the assistant clinician.

The above experimental results show that our deep learning model simultaneously extracts the spatial and temporal characteristics of the ECGs of patients with heart failure. The model focuses on the key features of the signals by incorporating the attention mechanism. These results show that the proposed model achieves a good classification result and that its comprehensive performance is better than similar methods.

## Conclusion

This paper proposes a deep learning model, CNN-LSTM-SE. The model uses a CNN, LSTM, and integrating attention mechanism. This model classifies heart failure into four levels automatically according to the ECG data of patients with heart failure.

We used a CNN to extract the spatial characteristics of ECGs. LSTM obtained the time series characteristics of ECGs. The attention mechanism was incorporated into the model to focus on the key features of ECGs to improve classification accuracy. We divided the ECGs into fragments of different lengths to construct the corresponding datasets and then assessed the model performance of different partitioning methods on the datasets. The datasets constructed with 12 s ECG signal segmentation provided the best classification with the proposed model. The comprehensive performance of the deep learning model described in this paper is better than the current shallow machine learning and similar deep learning models. It can assist medical staff in clinical diagnosis and has good application prospects. In medicine, all kinds of heart diseases need to process and analyze ECGs [34–37]. Therefore, this method is not limited to the field of heart failure classification, but can also be extended to other fields such as arrhythmia [38–40] and coronary artery disease [41–44].

The limitations of our CNN-LSTM-SE model are as follows:

1. The ECG segments input by the model should contain at least one complete ECG beat (P wave, PR segment [45–47], QRS complex, ST-T segment, U wave) to ensure more accurate classification results of the model. From the interpretability visualization results of the model, it can be known that if the input ECG segment does not contain a complete ECG beat, it may lead to the loss of some important features associated with four grades of heart failure, which affects the decision results of the model.
2. Our model belongs to the monomodal method based on ECGs for heart failure classification, without considering other clinical health data of heart failure patients, and there is still room for improvement in classification performance.

The further work based on the proposed model are as follows:

1. The proposed model is developed using imbalance dataset, we will work with hospitals to improve existing datasets, especially by adding data for NYHA Class I patients, to further refine the model’s performance.
2. Multimodal [48] network will be constructed to classify heart failure. On the basis of the deep learning model based on monomodal data in this paper, patient data from other modalities related to heart failure will be added to further improve the objectivity of heart failure classification results and the interpretability of related diseases. For example, adding clinical indicators such as blood pressure and blood glucose of patients to the model proposed in this paper can further explore the relationship between heart disease and underlying diseases [49] (such as hypertension, hyperglycemia, etc.).

## Data Availability

The MIMIC-III clinical dataset used in this study can be found in the Research Resource for Complex Physiologic Signals (PhysioNet), https://physionet.org/content/mimiciii/1.4/.The MIMIC-III waveform dataset used in this study can be found in the PhysioNet, https://physionet.org/content/mimic3wdb-matched/1.0/.All our source codes are available by contacting the corresponding author or first author.

## References

[1] Bredy C, Ministeri M, Kempny A, Alonso-Gonzalez R, Swan L, Uebing A, Diller G-P, Gatzoulis MA, Dimopoulos K (2018). New York heart association (NYHA) classification in adults with congenital heart disease: relation to objective measures of exercise and outcome. Eur Heart J-Qual Care Clin Outcomes.

[2] Chan ADC, Hamdy MM, Badre A, Badee V. Person Identification using Electrocardiograms. In: 2006 Canadian Conference on Electrical and Computer Engineering. 2006;1–4. https://doi.org/1-4.10.1109/CCECE.2006.277291.

[3] Aswath GI, Vasudevan SK, Sampath N (2020). A frugal and innovative telemedicine approach for rural India – automated doctor machine. Int J Med Engs Inform..

[4] Gupta V (2023). Wavelet transform and vector machines as emerging tools for computational medicine. Journal of ambient intelligence and humanized. Computing..

[5] Belderrar A, Hazzab A (2020). Real-time estimation of hospital discharge using fuzzy radial basis function network and electronic health record data. Int J Med Eng Inform..

[6] Ramachandran SK, Manikandan P (2020). An efficient ALO-based ensemble classification algorithm for medical big data processing. Int J Med Eng Inform..

[7] Balasubramanian K, Ananthamoorthy NP (2021). Robust retinal blood vessel segmentation using convolutional neural network and support vector machine. J Ambient Intell Humaniz Comput..

[8] Tripoliti EE, Papadopoulos TG, Karanasiou GS, Kalatzis FG, Bechlioulis A, Goletsis Y, Naka KK, Fotiadis DI. Estimation of New York Heart Association class in heart failure patients based on machine learning techniques. In: 2017 IEEE EMBS International Conference on Biomedical & Health Informatics (BHI). 2017;421–424. 10.1109/BHI.2017.7897295.

[9] Zhang R, Ma S, Shanahan L, Munroe J, Horn S, Speedie S. Discovering and identifying New York heart association classification from electronic health records. Med Inform Decision Making. 2018;18(2):5–13. 10.1186/s12911-018-0625-7. 10.1186/s12911-018-0625-7 PMC6069768 30066653

[10] Qu Z, Liu Q, Liu C (2019). Classification of congestive heart failure with different New York heart association functional classes based on heart rate variability indices and machine learning. Expert Syst..

[11] Li DG, Li X, Zhao JM, Bai XH (2019). Automatic staging model of heart failure based on deep learning. Biomed Signal Proces..

[12] Li D, Tao Y, Zhao J, Wu H (2020). Classification of congestive heart failure from ECG segments with a multi-scale residual network. Symmetry-Basel..

[13] D'Addio G, Donisi L, Cesarelli G, Amitrano F, Coccia A, La Rovere MT, Ricciardi C (2021). Extracting features from Poincare plots to distinguish congestive heart failure patients according to NYHA classes. Bioengineering-Basel..

[14] Sandhu JK, Lilhore UK, Poongodi M, Kaur N, Band SS, Hamdi M, Iwendi C, Simaiya S, Kamruzzaman MM, Mosavi A. Predicting the risk of heart failure based on clinical data. HCIS. 2022;12 10.22967/HCIS.2022.12.057.

[15] Tsai IH, Morshed BI. Beat-by-beat Classification of ECG Signals with Machine Learning Algorithm for Cardiac Episodes. In: 2022 IEEE International Conference on Electro Information Technology (eIT). 2022;311–314. 10.1109/eIT53891.2022.9813902.

[16] Mokeddem F, Meziani F, Debbal SM (2020). Study of murmurs and their impact on the heart variability. Int J Med Eng Inform..

[17] Johnson AEW, Pollard TJ, Shen L, L-wH L, Feng M, Ghassemi M, Moody B, Szolovits P, Celi LA, Mark RG (2016). MIMIC-III, a freely accessible critical care database. Sci Data..

[18] Zhou F, Yang S, Fujita H, Chen D, Wen C (2020). Deep learning fault diagnosis method based on global optimization GAN for unbalanced data. Knowl-Based Syst..

[19] Admass WS, Bogale GA. Arrhythmia classification using ECG signal: a meta-heuristic improvement of optimal weighted feature integration and attention-based hybrid deep learning model. Biomed Signal Proces. 2024;87:–105565. 10.1016/j.bspc.2023.105565.

[20] Acharya UR, Fujita H, Oh SL, Hagiwara Y, Tan JH, Adam M (2017). Application of deep convolutional neural network for automated detection of myocardial infarction using ECG signals. Inform Sci..

[21] Wang H, Liu Z, Peng D, Qin Y. Understanding and learning discriminant features based on multiattention 1DCNN for wheelset bearing fault diagnosis. IEEE Trans Industr Inform. 2020; 16(9):5735–5745.10.1109/TII.2019.2955540.

[22] Lecun Y, Bottou L, Bengio Y, Haffner P (1998). Gradient-based learning applied to document recognition. Proc IEEE..

[23] Greff K, Srivastava RK, Koutnik J, Steunebrink BR, Schmidhuber J (2017). LSTM: a search space odyssey. IEEE Trans Neural Netw Learn Syst..

[24] Gers FA, Schmidhuber J, Cummins F (2000). Learning to forget: continual prediction with LSTM. Neural Comput..

[25] Hu J, Shen L, Albanie S, Sun G, Wu E (2020). Squeeze-and-excitation networks. IEEE Trans Pattern Anal Mach Intell..

[26] Kiritchenko S, Zhu X, Mohammad SM (2014). Sentiment analysis of short informal text. J Artif Intell Res..

[27] Duda RO, Hart PE, Stork DG (2001). Pattern classification.

[28] Baim DS, Colucci WS, Monrad ES, Smith HS, Wright RF, Lanoue A, Gauthier DF, Ransil BJ, Grossman W, Braunwald E (1986). Survival of patients with severe congestive heart failure treated with oral milrinone. J Am Coll Cardiol..

[29] Iyengar N, Peng CK, Morin R, Goldberger AL, Lipsitz LA (1996). Age-related alterations in the fractal scaling of cardiac interbeat interval dynamics. Am J Physiol..

[30] Couderc J-P. The Telemetric and Holter ECG Warehouse (THEW): The first three years of development and research. J Electrocardiol. 2012;45(6):677–83. 10.1016/j.jelectrocard.2012.08.001. 10.1016/j.jelectrocard.2012.08.001 PMC3483367 23022305

[31] Acharya UR, Fujita H, Oh SL, Hagiwara Y, Tan JH, Adam M, Tan RS (2019). Deep convolutional neural network for the automated diagnosis of congestive heart failure using ECG signals. Appl Intell..

[32] Zhang CJ, Wang XJ, Ma LM, Lu XQ (2021). Tropical cyclone intensity classification and estimation using infrared satellite images with deep learning. IEEE J Sel Top Appl Earth Obs Remote Sens..

[33] Hendry PB, Krisdinarti L, Erika M (2016). Scoring system based on electrocardiogram features to predict the type of heart failure in patients with chronic heart failure. Cardiol Res..

[34] Gupta V, Mittal M, Mittal V, Saxena NK (2022). Spectrogram as an emerging tool in ECG signal processing. Recent advances in manufacturing, automation, design and energy technologies: 2022// 2022.

[35] Gupta V, Mittal M, Mittal V, Gupta A (2021). An efficient AR modelling-based electrocardiogram signal analysis for health informatics. Int J Med Eng Inform..

[36] Gupta V, Mittal M (2019). QRS complex detection using STFT, Chaos analysis, and PCA in standard and real-time ECG databases. J Inst Eng (India): Series B..

[37] Gupta V, Mittal M, Mittal V, Diwania S, Saxena NK (2023). ECG signal analysis based on the spectrogram and spider monkey optimisation technique. J Inst Eng (India): Series B..

[38] Gupta V (2022). Application of chaos theory for arrhythmia detection in pathological databases. Int J Med Eng Inform..

[39] Gupta V, Mittal M, Mittal V (2021). Chaos theory and ARTFA: emerging tools for interpreting ECG signals to diagnose cardiac arrhythmias. Wirel Pers Commun..

[40] Gupta V, Mittal M, Mittal V (2022). A novel FrWT based arrhythmia detection in ECG signal using YWARA and PCA. Wirel Pers Commun..

[41] Li S, Nunes JC, Toumoulin C, Luo L (2018). 3D coronary artery reconstruction by 2D motion compensation based on mutual information. IRBM..

[42] Mabrouk S, Oueslati C, Ghorbel F (2017). Multiscale graph cuts based method for coronary artery segmentation in angiograms. IRBM..

[43] Harmouche M, Maasrani M, Verhoye JP, Corbineau H, Drochon A (2014). Coronary three-vessel disease with occlusion of the right coronary artery: what are the most important factors that determine the right territory perfusion?. IRBM..

[44] Velut J, Lentz PA, Boulmier D, Coatrieux JL, Toumoulin C (2011). Assessment of qualitative and quantitative features in coronary artery MRA. IRBM..

[45] Gupta V, Saxena NK, Kanungo A, Kumar P, Diwania S (2022). PCA as an effective tool for the detection of R-peaks in an ECG signal processing. Int J Syst Assur Eng Manag..

[46] Gupta V, Mittal M, Mittal V (2021). FrWT-PPCA-based R-peak detection for improved Management of Healthcare System. IETE J Res..

[47] Gupta V, Mittal M, Mittal V, Chaturvedi Y (2022). Detection of R-peaks using fractional Fourier transform and principal component analysis. J Ambient Intell Humaniz Comput..

[48] Xu X, Huang L, Wu R, Zhang W, Ding G, Liu L, Chi M, Xie J (2022). Multi-feature fusion method for identifying carotid artery vulnerable plaque. IRBM..

[49] Helen MMC, Singh D, Deepak KK (2020). Changes in scale-invariance property of electrocardiogram as a predictor of hypertension. Int J Med Eng Inform..

